# Evaluating sowing uniformity in hybrid rice using image processing and the OEW-YOLOv8n network

**DOI:** 10.3389/fpls.2025.1473153

**Published:** 2025-02-03

**Authors:** Zehua Li, Yihui Pan, Xu Ma, Yongjun Lin, Xicheng Wang, Hongwei Li

**Affiliations:** ^1^ College of Mathematics and Informatics, South China Agricultural University, Guangzhou, China; ^2^ Key Laboratory of Smart Agricultural Technology in Tropical South China, Ministry of Agriculture and Rural Affairs, Guangzhou, China; ^3^ College of Engineering, South China Agricultural University, Guangzhou, China; ^4^ School of Mechanical Engineering, Guangxi University, Nanning, China

**Keywords:** mechanical sowing, uniformity evaluation, deep learning, object detection, rice seeder

## Abstract

Sowing uniformity is an important evaluation indicator of mechanical sowing quality. In order to achieve accurate evaluation of sowing uniformity in hybrid rice mechanical sowing, this study takes the seeds in a seedling tray of hybrid rice blanket-seedling nursing as the research object and proposes a method for evaluating sowing uniformity by combining image processing methods and the ODConv_C2f-ECA-WIoU-YOLOv8n (OEW-YOLOv8n) network. Firstly, image processing methods are used to segment seed image and obtain seed grids. Next, an improved model named OEW-YOLOv8n based on YOLOv8n is proposed to identify the number of seeds in a unit seed grid. The improved strategies include the following: (1) Replacing the Conv module in the Bottleneck of C2f modules with the Omni-Dimensional Dynamic Convolution (ODConv) module, where C2f modules are located at the connection between the Backbone and Neck. This improvement can enhance the feature extraction ability of the Backbone network, as the new modules can fully utilize the information of all dimensions of the convolutional kernel. (2) An Efficient Channel Attention (ECA) module is added to the Neck for improving the network’s capability to extract deep semantic feature information of the detection target. (3) In the Bbox module of the prediction head, the Complete Intersection over Union (CIoU) loss function is replaced by the Weighted Intersection over Union version 3 (WIoUv3) loss function to improve the convergence speed of the bounding box loss function and reduce the convergence value of the loss function. The results show that the mean average precision (mAP) of the OEW-YOLOv8n network reaches 98.6%. Compared to the original model, the mAP improved by 2.5%. Compared to the advanced object detection algorithms such as Faster-RCNN, SSD, YOLOv4, YOLOv5s YOLOv7-tiny, and YOLOv10s, the mAP of the new network increased by 5.2%, 7.8%, 4.9%, 2.8% 2.9%, and 3.3%, respectively. Finally, the actual evaluation experiment showed that the test error is from −2.43% to 2.92%, indicating that the improved network demonstrates excellent estimation accuracy. The research results can provide support for the mechanized sowing quality detection of hybrid rice and the intelligent research of rice seeder.

## 1 Introduction

Hybrid rice is an important food crop, accounting for over 50% of China’s rice planting area. It has been planted in over 70 countries worldwide, with a cumulative planting area exceeding 600 million ha, making significant contributions to global food security (Ma and Yuan, 2015; Yuan, 2018). However, the quality of mechanical sowing of hybrid rice is one of the important factors restricting the development of hybrid rice production (Chen et al., 2015; Li et al., 2018; Ma et al., 2023) where sowing uniformity is an important evaluation indicator of the quality of mechanized sowing (Alekseev et al., 2018). Currently, the evaluation of the sowing uniformity in hybrid rice mainly relies on manual visual inspection, which is highly subjective. In order to achieve an objective evaluation of sowing uniformity, provide timely feedback on the sowing quality information to the performance control system of the seeders, and effectively improve sowing quality, this paper combines image processing methods and deep learning algorithms to study a method for evaluating the sowing uniformity of hybrid rice blanket-seedling nursing.

In the mechanized seedling nursing of hybrid rice, according to the different types of seedling trays, seedling nursing methods are divided into pot-seedling nursing and blanket-seedling nursing, where pot trays and blanket trays are used, respectively. The pot tray is a type of plastic tray. It is usually composed of 406 (29 rows×14 columns) or 448 (32 rows×14 columns) pots arranged uniformly. The blanket tray is also a type of plastic tray, which is essentially an uncovered cuboid with a typical inner cavity size of 25 mm × 280 mm × 580 mm. When a pot tray is used for sowing, the seeds in the tray are separated into relatively separate seed groups by pots, while when a blanket tray is used for sowing, the seeds in the tray are relatively uniformly arranged, but there is still a certain degree of randomness in the direction and position of the seeds. This indicates that compared to blanket-seedling nursing, the target area of sowing quality inspection for pot-seedling nursing is relatively easy to be determined. Therefore, both domestically and internationally, research on the sowing quality detection of hybrid rice mechanized sowing has mainly focused on pot-seedling nursing (Qi et al., 2009; Zhou et al., 2012; Zhao et al., 2014; Wang et al., 2015; Chen et al., 2017; Wang et al., 2018), in which the combination of image processing and machine vision technology is mainly used. There are relatively fewer reports on the sowing quality detection of blanket-seedling nursing, and only articles published by Tan et al. (2014) and Dong et al. (2020) have been found. Among them, Tan et al. (2014) established a BP neural network model for detecting the sowing quantity of super hybrid rice pot-seedling nursing by extracting shape features such as area, perimeter, and shape factor of the seed-connected region. The average accuracy of detection has reached 94.4%. Dong et al. (2020) designed a sowing quantity detection and control device for hybrid rice based on embedded machine vision, where the algorithm for detecting the sowing quantity is similar to the algorithm in Tan et al. (2014).

In recent years, deep learning has demonstrated excellent performance in complex scene object detection and has been widely applied in agricultural production (Johansen et al., 2019a, b, 2020; Jiang et al., 2022; Yun et al., 2024). Although no studies using deep learning methods to investigate the sowing uniformity of hybrid rice blanket-seedling nursing have been found, deep learning methods have been applied for uniformity detection in other crop production, including tasks such as corn seedling emergence uniformity detection. For instance, Nee et al. (2022) utilized UAV imagery and deep learning model ResNet18 to estimate and map corn emergence uniformity. Corn emergence uniformity was quantified with plant density, plant spacing standard deviation, and mean days to imaging after emergence.

You Only Look Once (YOLO) is a typical representative of one-stage networks in deep learning, characterized by high detection accuracy and fast speed, and has been widely used in non-contact object detection. For example, Wang et al. (2023) proposed an improved YOLOv5 model named CM-YOLOv5s-CSVPoVnet model for yellow peach detection by adding model clipping, Omni-Dimensional Dynamic Convolution (ODConv), Global Context Networks, and ELAN methods. The results showed that the mean average precision (mAP) reached 96%, and the model size was 3.54 MB. Zhang et al. (2023) enhanced the YOLOv5s model by incorporating the Efficient Channel Attention (ECA)-Net attention mechanism module and Adaptively Spatial Feature Fusion (ASFF) into the feature pyramid structure of YOLO, thus creating the YOLOv5-ECA-ASFF model. This model was utilized for detecting wheat scab fungus spores, and the results demonstrated that the average recognition accuracy reached 98.57%. The YOLO series has algorithms such as YOLOv1 to YOLOv10, with the characteristic that newer versions are more advanced. YOLO has demonstrated excellent detection performance in the above studies, being able to quickly count the number of targets, which is very enlightening for detecting the sowing uniformity of hybrid rice.

In this paper, according to the characteristics of sowing quality detection of hybrid rice blanket-seedling nursing, an improved algorithm named ODConv_C2f-ECA-WIoU-YOLOv8n (OEW-YOLOv8n) network is proposed based on the YOLOv8n algorithm for identifying the number of seeds in a unit seed grid. Then, combining image processing methods with the OEW-YOLOv8n network, a method for evaluating sowing uniformity of hybrid rice blanket-seedling nursing is promoted. The sowing uniformity of detection in seed image has been implemented on computer devices. The relevant results can provide support for detecting the sowing quality of hybrid rice.

## 2 Materials and methods

### 2.1 Mechanical sowing and image acquisition

**Figure 1:**
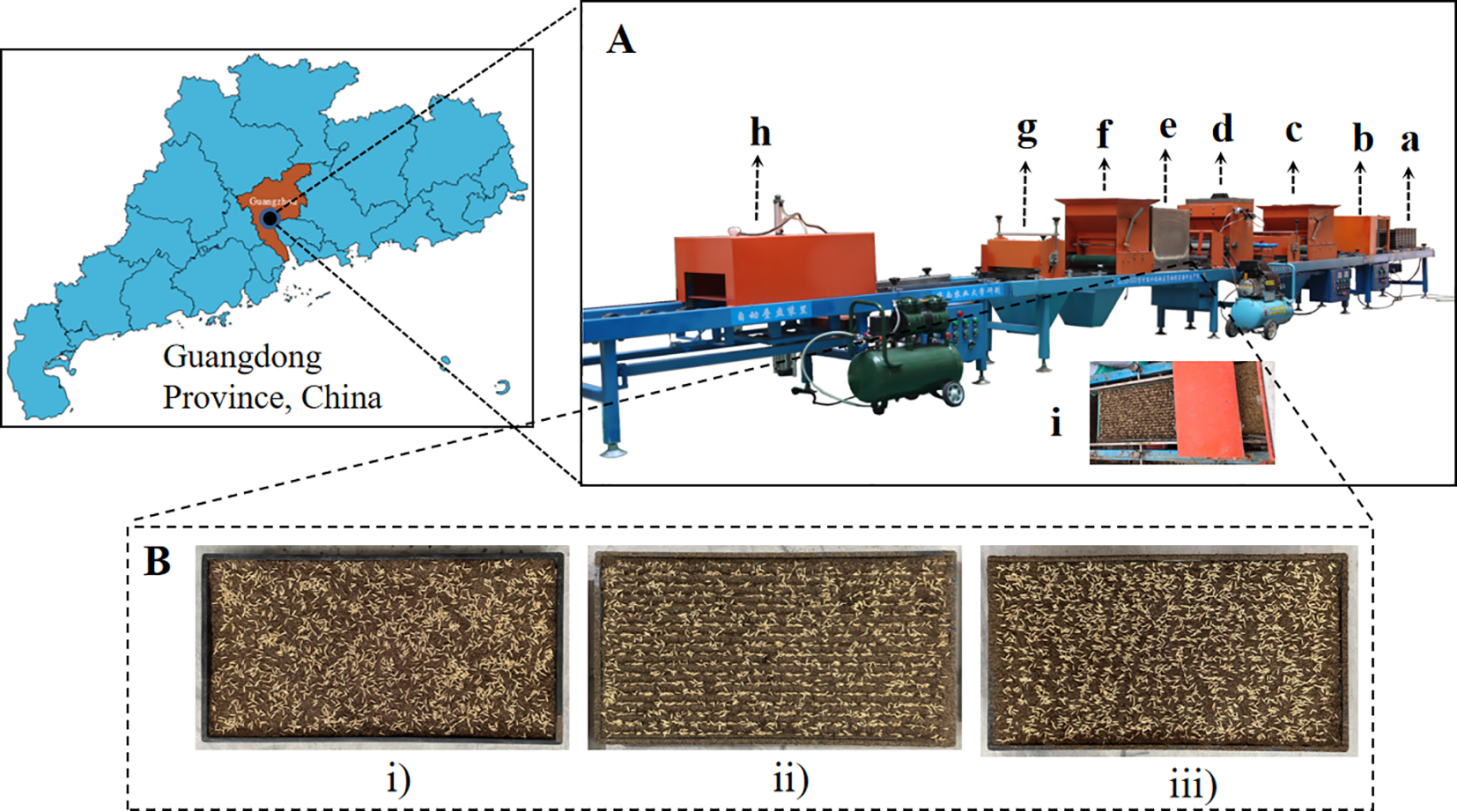
Representative seed images and mechanical sowing process. **(A)** demonstrates the mechanical sowing process, where a-i sequentially represents the seedling tray, automatic laying tray, laying bottom soil, precision sowing, laying topsoil, cleaning topsoil, automatic stacking tray, and precision sowing. In **(B)**, the seed images are shown, where i, ii, and iii represent broadcast sowing with 50 g/tray, ditching strip sowing with 60 g/tray, and no-ditching strip sowing with 70 g/tray, respectively.

The mechanical sowing experiment was conducted on 1 July 2023 at the Soil Trough Laboratory of College of Engineering, South China Agricultural University, Guangdong Province (latitude 23°16′, longitude 113°35′). The time for mechanical sowing is from 9:00 a.m. to 10:00 a.m., and the time for image acquisition is from 10:00 a.m. to 12:00 p.m. The used seeder is the 2ZSB-500 intelligent rice tray seedling precise seeding production line developed by South China Agricultural University. The experimental variety is hybrid rice taifengyou 208. Before mechanical sowing, the seeds are carefully selected and germinated. The seedling trays are standard hard blanket-seedling trays with specifications of 25 mm × 280 mm × 580 mm. The mechanical sowing process mainly includes laying tray, laying bottom soil, and sowing. After sowing, the seeds are not covered by topsoil for capturing seed images. The seedling trays with seeds are placed on a flat ground. Under natural light conditions (during the experiment, the strength of illumination was between 10,000 and 30,000 lux), the seed images of the entire seedling tray were acquired by a smartphone fixed on a simple camera perch. The smartphone is an Apple iPhone 12, with a resolution of 2,268 × 4,032 pixels. In order to improve the generalization ability of the model by increasing the diversity of target data, the sowing methods include broadcast sowing, ditching strip sowing, and no-ditching strip sowing, and the sowing density consists of three levels: 50 g/tray, 60 g/tray, and 70 g/tray. Thus, there are nine kinds of sowing modes. For each sowing mode, 5 trays were sown, resulting in a total of 45 trays having been sown. For each tray, five photographs with different shooting effects were selected. Finally, we obtained 225 valid photographs. Among them, 18 photographs were used as training samples, consisting of 2 photographs of trays from 9 sowing modes. The remaining 207 photographs were used as application samples to evaluate sowing uniformity. The representative seed images and mechanical sowing process are shown in **Figure 1**.

### 2.2 The main process of evaluating sowing uniformity

**Figure 2:**
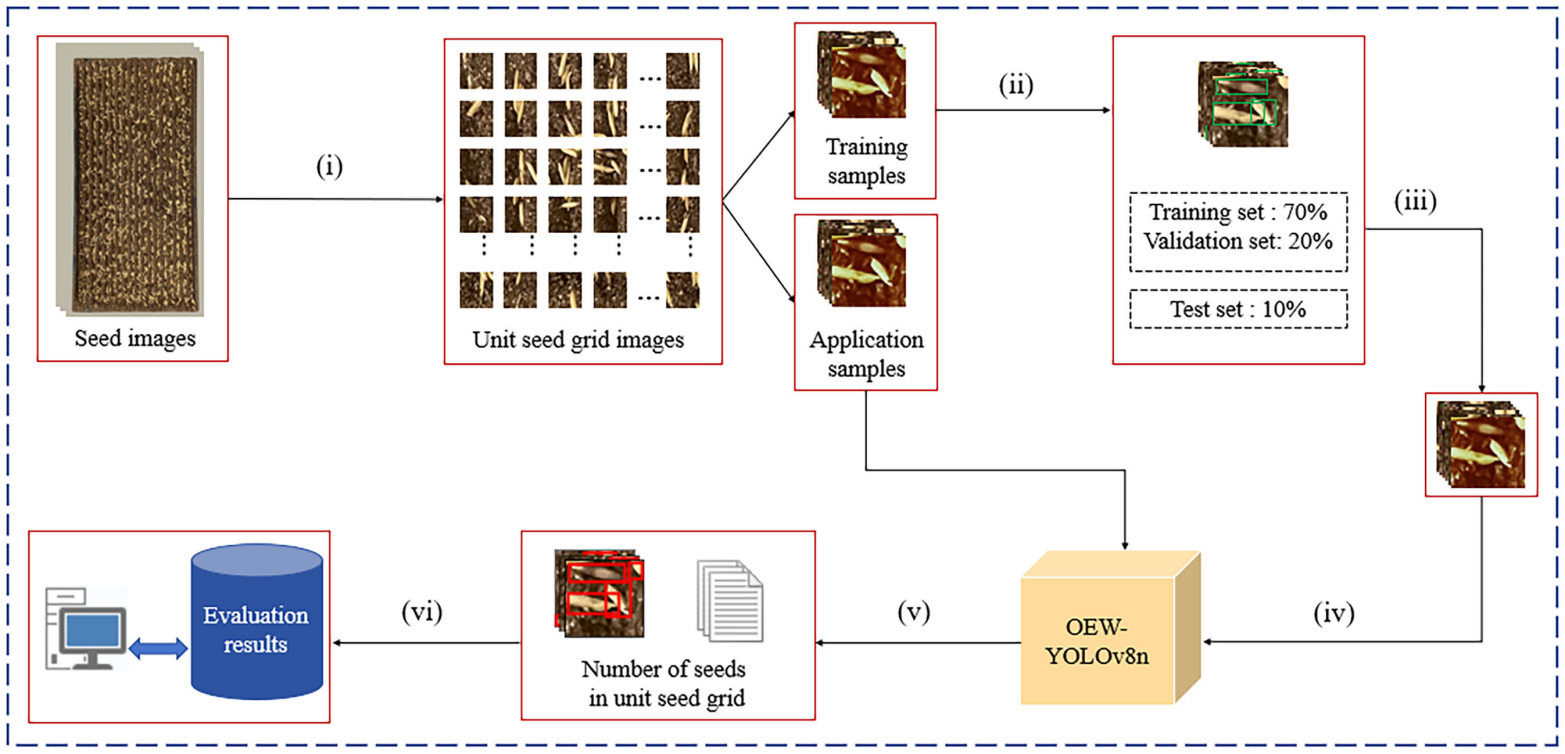
The main process of evaluating sowing uniformity. (i) image preprocessing (ii) labeling (iii) data augmentation (iv) model training and testing (v) detect unit seed grid images (vi) evaluate uniformity Modification of problem.

**Figure 3:**
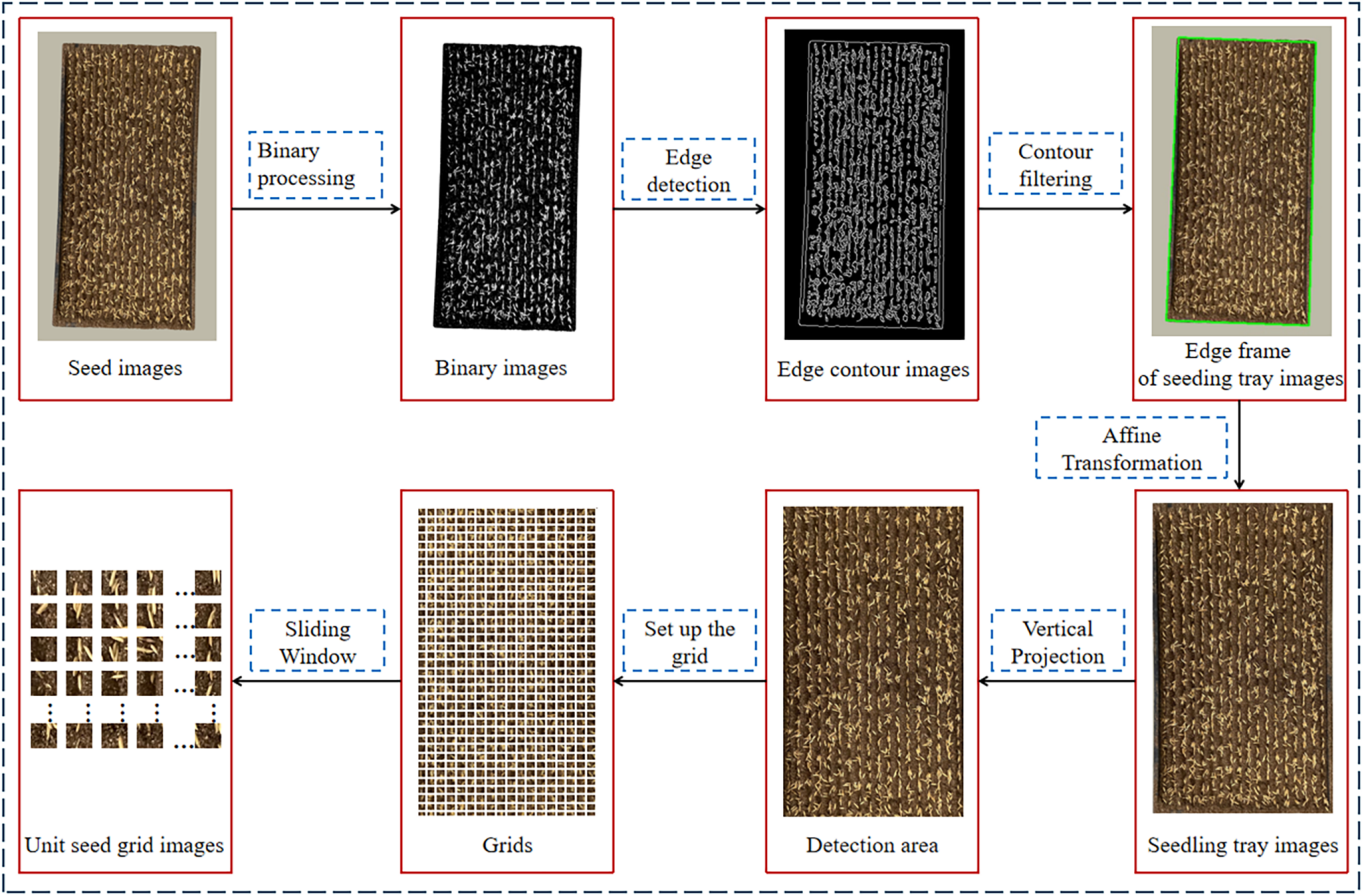
Image preprocessing.

The evaluation of sowing uniformity mainly includes three parts: image preprocessing, model training, and uniformity evaluation, as shown in **Figure 2**. Image preprocessing is shown in **Figure 3**.

The image preprocessing process is as follows: (1) Inputting seed images and obtaining binary images by using binarization. (2) Using Canny operator to perform edge detection on binary images and obtain edge contour images. (3) Using contour filtering to obtain the edge frame of seedling tray images. (4) Correcting the graphics through affine transformation to obtain seedling tray images, which includes detection area and the borders of seedling tray. (5) Locating the detection area through the vertical projection method (see Section 2.3.1). (6) Setting the number of grids, use the Sliding Window method to segment the detection area and obtain unit seed grid images (see Section 2.3.2).

The model training mainly includes (1) dividing the unit seed grid images into training samples and application samples, (2) using the LabelImg software to label the seeds in training samples, (3) using image augmentation methods to expand the training set, and (4) inputting the dataset into the OEW-YOLOv8n model for training and testing.

The uniformity evaluation part mainly includes the following: The seed images of the application samples are treated undergoing the image preprocessing process, and the seed grid images of the application samples will be obtained. Then, input the seed grid images of the application samples into the OEW-YOLOv8n model for detection, and the number of seeds per grid will be obtained. Finally, calculate the qualification rates of grids of one to three seeds per grid to evaluate the sowing uniformity.

### 2.3 Methods for detection area localization and seed grid segmentation based on image processing

#### 2.3.1 Detection area localization algorithm

Because the seedling tray image includes some non-detection areas such as the border of the seedling tray, it is necessary to locate the detection area. The vertical projection method (Chen et al., 2021) is used to solve this problem in this paper.

**Figure 4:**
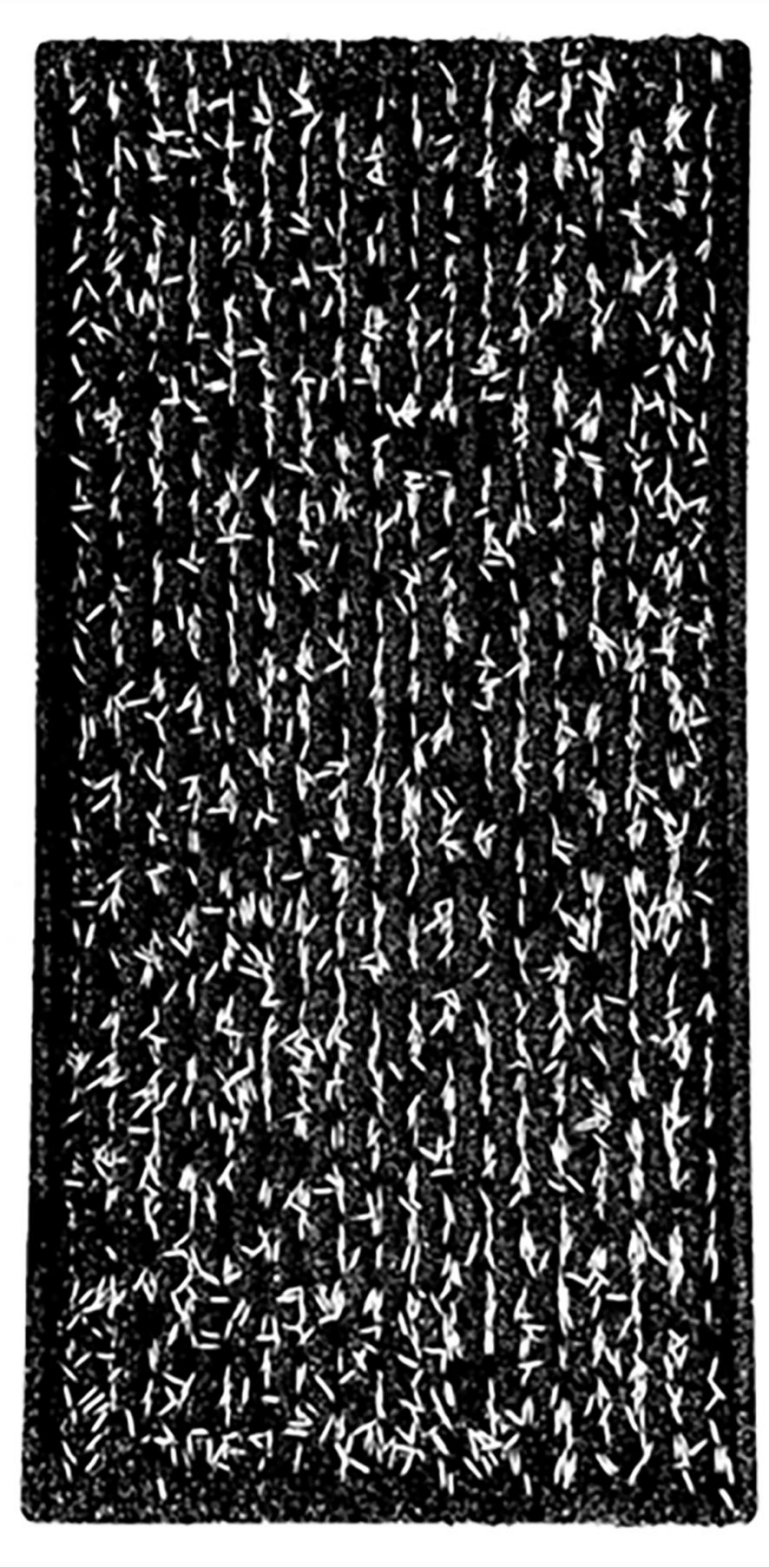
Binary seedling tray image.


The vertical projection method is as follows: In the binary seedling tray image (**Figure 4**), white oval dots represent seeds with a grayscale value of 0, while black areas represent non-seed parts such as seedling soil or the border of the seedling tray, with a grayscale value of 1. The cumulative grayscale values for each row and each column are computed in pixel units, following the methods described in Equations 1 and 2.

(1) $$w(i)=\sum_{j=0}^{H-1}p(i,j),i=0,1,\ldots ,W-1$$

(2) $$h(j)=\sum_{i=0}^{W-1}p(i,j),j=0,1,\ldots ,H-1$$

where *W* and *H* represent the number of rows and columns, respectively. In this paper, *W* = 4,032 and *H* = 2,268. *w*(*i*) represents the cumulative grayscale value of the *i*th row, *h*(*j*) represents the cumulative grayscale value of the *j*th column, and *p*(*i*,*j*) represents the grayscale value of the corresponding pixel at the *i*th row and *j*th column.

**Figure 5:**
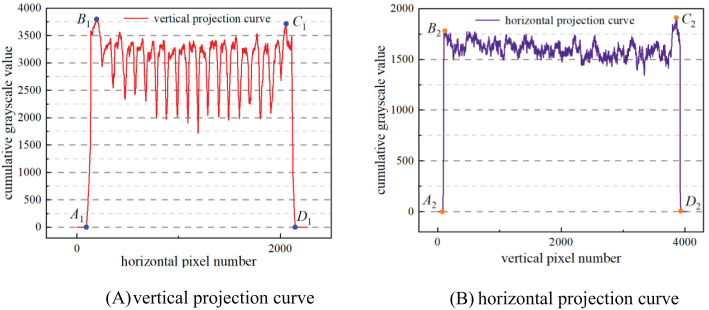
Projection curve. **(A)** vertical projection curve **(B)** horizontal projection curve.

Based on the grayscale values of the binary image, a vertical projection curve (**Figure 5A**) was plotted with the number of horizontal pixels as abscissa and the cumulative grayscale value of each column as ordinate. Similarly, a horizontal projection curve (**Figure 5B**) was plotted with the number of vertical pixels as abscissa and the cumulative grayscale value of each row as ordinate. As shown in **Figure 5**, there are four abrupt segments on both sides of the images of the vertical projection curve and the horizontal projection, namely, A_1_B_1_, C_1_D_1_, or A_2_B_2_, C_2_D_2_. In segments A_1_B_1_ and A_2_B_2_, the cumulative grayscale value suddenly increases from 0 to a larger value, owing to the appearance of the border of the seedling tray in the image, in which A_1_ and A_2_ represent the outer side of the seedling tray border, while B_1_ and B_2_ represent the inner side of the seedling tray border. Conversely, in segments C_1_D_1_ and C_2_D_2_, the cumulative grayscale value suddenly drops from a larger value to 0. The reason for this is that the border of the seedling tray in the image disappears. At this point, C_1_ and C_2_ represent the inside of the seedling tray border, while D_1_ and D_2_ represent the outside of the seedling tray border. This indicates that A_1_B_1_, C_1_D_1_, A_2_B_2_, and C_2_D_2_ respectively represent the positions of the four borders of the seedling tray. Taking the positions of the pixel numbers corresponding to B_1_, C_1_, B_2_, and C_2_ as the positions of the four edge lines of the detection area, then the detection area is located.

#### 2.3.2 Seed grid segmentation method

In general, in order to distinguish the description of pots in a pot tray, it is customary to refer to the unit area of blanket tray as the seed grid. A seed grid in a blanket tray is equivalent to one pot in the pot tray. In order to evaluate the sowing uniformity of blanket-seedling nursing, it is necessary to manually divide seed grids and mark them in seed images.

Without considering the deformation of seedling blocks, one seed grid is equal to the unit seedling-taken area during mechanical transplanting. A unit seedling-taken area of mechanical transplanting = horizontal seedling cutting quantity × longitudinal seedling cutting quantity. Therefore, the key to dividing seed grids is to determine the horizontal seedling cutting quantity and the longitudinal seedling cutting quantity. In theory, the number of seeds in the seed grid is the basis for estimating the number of seedlings per hill in the field during mechanical transplanting. Practical experience shows that the actual germination rate of hybrid rice seeds is from 75% to 85% (Li et al., 2019). To achieve high-yield cultivation of hybrid rice, theoretically, the number of seeds in one seed grid is relatively suitable at 2.7–2.9. At this time, the average number of seedlings per hill is 2–2.5. Therefore, the method for dividing seed grids in seed images is as follows:

In hybrid rice blanket-seedling nursing with strip sowing, an 18-row seeder is usually used for sowing. After sowing, the seeds in the seedling tray appear as 18 seed strips. Therefore, the seed image is evenly divided into 18 parts horizontally. At this time, the number of cutting times of horizontal seedling by seedling claw of the rice transplanter is 18, and the horizontal seedling cutting quantity is 15.6 mm (208 mm ÷ 18 = 15.6 mm). The number of cutting times of longitudinal seedling by seedling claw of the rice transplanter is determined by the longitudinal seedling cutting quantity. The longitudinal seedling cutting quantity is set by the longitudinal seedling feeding mechanism of the transplanter. Usually, the adjustment range for the longitudinal seedling feeding mechanism is from 8 to 18 mm. There are 11 gears available, with a 1-mm interval between each gear.

In order to achieve the target of precise transplanting of one to three seedlings per hill, the number of seed grids per tray is related to the sowing density. The higher the sowing density, the more the seed grids there will be. According to the thousand-grain weight and sowing density of hybrid rice seeds, the number and the division method of seed grids under different sowing densities are calculated as shown in **Table 1**.

**Table 1:** Number of seed grids in a seedling tray and division method under different sowing densities.

Sowing density/(g·tray−1)	50	60	70
The number of cutting times of horizontal seedling by seedling claw of the rice transplanter/times	18	18	18
Longitudinal seedling cutting quantity/mm	14	12	10
The number of cutting times of longitudinal seedling by seedling claw of the rice transplanter/times	41	48	58
Theoretically, the number of seed grids per tray/grid	738	864	1,044
Theoretically, the number of seeds per tray/grain	2,020	2,424	2,828
Theoretically, the number of seeds per grid/grain	2.74	2.81	2.71

In **Table 1**, the thousand-grain weight of hybrid rice taifengyou 208 is 24.75 g. When the sowing density is 50 g/tray, theoretically, there are approximately 2,020 seeds per tray. If the number of seed grids per tray is set to 738 grids (18 rows × 41 columns = 738 grids), then the average number of seeds per grid is 2.74, which meets the agronomic requirements. Similarly, when the sowing density is 60 g/tray and 70 g/tray, the seed grids per tray is 864 grids (18 rows × 48 columns) and 1,044 grids (18 rows × 58 columns), respectively. Theoretically, the number of seeds per grid is approximately 2.81 and 2.71, respectively.

### 2.4 Dataset creation

After localizing the detection areas of the training samples for the model, we get 18 seed images only containing detection areas. According to the seed grid division methods of seedling tray under different sowing densities in **Table 1**, using the Sliding Window method to divide the seed images, we obtained 15,876 unit seed grid images [(738 + 864 + 1,044) × 3 × 2 = 15,876].

This paper uses the LabelImg tool to label seeds in seed grid images, naming the labels “seed” and generating corresponding txt label files. Expand the training sample dataset using data augmentation methods, which mainly include cropping, adding Gaussian noise, horizontal flipping, vertical flipping, color transformation, contrast transformation, and randomly scaling the width and height of seed grid images within a reasonable range. The expanded training dataset includes 23,814 images with a total of 82,196 seed labels. The expanded images and their corresponding text label files are randomly divided into a training set and a testing set in a 7:2:1 ratio (as shown in **Table 2**).

**Table 2:** Distribution of seed grid images for training sample.

Categories	Number of images	Number of labels
Training set	16,669	57,535
Validation set	4,763	16,441
Test set	2,382	8,220

### 2.5 OEW-YOLOv8n model

YOLOv8 is an updated version of YOLOv5 developed by Ultralytics company. Compared to the previous-generation YOLOv5, the main improvements in YOLOv8 include the following: (1) In the Backbone network, the C3 module has been replaced by the C2f module, achieving further lightweight; (2) in the Neck, the Path Aggregation Network–Feature Pyramid Network (PA-FPN) concept has been adopted, removing the convolution operations during the upsampling process; (3) the Decoupled-Head structure is adopted in the Head, separating the classification and detection tasks, further reducing the complexity of the model; and (4) in the loss function, binary cross-entropy loss (BCE Loss) is used for classification, and distribution focal loss (DF Loss) along with Complete Intersection over Union (CIoU) Loss is used for regression. These improvements allow YOLOv8 to retain the advantages of the YOLOv5 network structure while making more refined adjustments and optimizations, enhancing the model’s performance in various scenarios. YOLOv8 is a family of models, ranging from smaller to larger versions, including YOLOv8n, YOLOv8s, YOLOv8m, YOLOv8l, and YOLOv8x. The key in evaluating sowing uniformity in hybrid rice blanket-seedling nursing is identifying the number of seeds in a unit seed grid. To ensure the model is lightweight, this study constructs the model based on YOLOv8n, which has the minimum number of model parameters.

The YOLOv8n network mainly consists of four parts: Input, Backbone, Neck, and Head. The Input terminal enhances the unit seed grid image through Mosaic augmentation before feeding it into the network. The Backbone network replaces the C3 module with the C2f module and uses the CSPDarkNet53 network to extract features from top to bottom. Additionally, it includes five Conv modules and one SPPF module. The C2f module contains two Conv modules, one split module, *n* Bottleneck modules, and one Concat operation. The C2f module captures rich gradient information from the feature map. The SPPF module uses convolutional kernels of different sizes to pool the feature map, obtaining multi-scale features. It then utilizes the Concat operation to achieve multi-scale feature fusion. The Neck network mainly consists of the PAN. PAN consists of the Feature Pyramid Network (FPN) and a bottom-up PAN. FPN passes rich semantic information from the top to the bottom, enhancing the semantic information of shallow feature maps, while the bottom-up PAN propagates strong localization feature information from the bottom up. The dual-pyramid structure of the Neck network further enhances the representation capability of multi-scale features. The prediction head outputs the classification and coordinate information of detected targets in three different size branches. The classification loss function uses BCE Loss, and the object detection regression loss functions use DF Loss and CIoU Loss.

**Figure 6:**
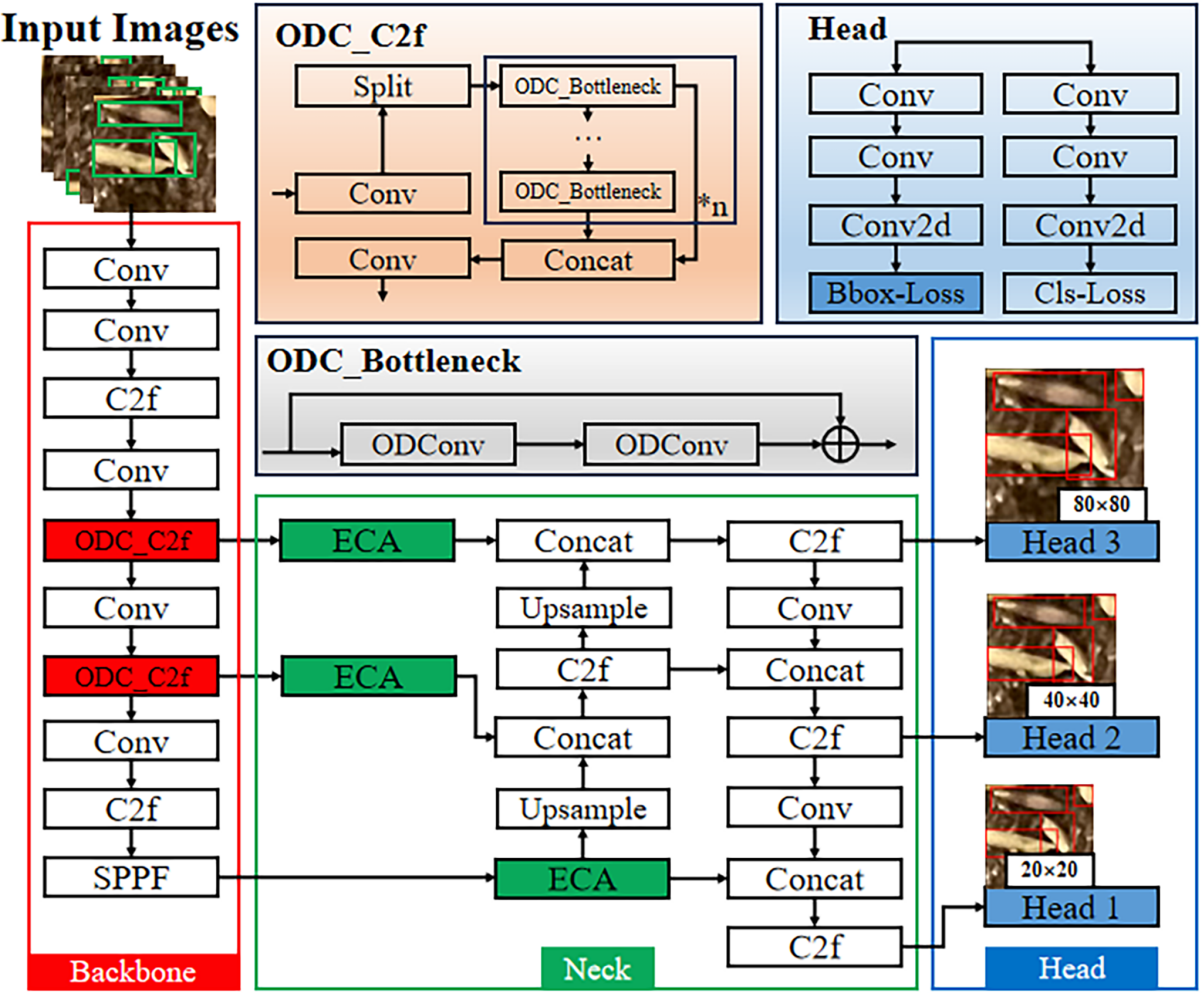
The architecture of OEW-YOLOv8n. The improved modules are highlighted in red, green, or blue colors, while the white boxes represent the original network structure modules. The improved Bottleneck module is named ODC_Bottleneck, and the improved C2f module is named ODC_C2f.

The aim of this study is to identify the number of seeds in a unit seed grid. Hybrid rice seed images have the following characteristics: (1) Each image contains a large number of seeds, with individual seeds occupying relatively few pixels, which falls under the category of small object detection. (2) Because of seed grid division, some seeds are artificially cut, so some seeds are incomplete in a unit seed grid, resulting in diversity of the targets. (3) Some seeds exhibit crossing, overlapping, or occlusion in the images. (4) The background of seed images is relatively complex for the nursery soil and consists of different components with different colors and shapes. Based on the above characteristics, this paper proposes the following improvements to YOLOv8n: (1) Adjust the C2f module connected to Neck in the Backbone structure and replace the Conv module in the Bottleneck structure with the ODConv module (Li et al., 2022), so that this module fully utilizes the information of all dimensions of the convolutional kernels, enhancing the feature extraction capability of the model’s Backbone network and improving the accuracy of small object detection. (2) Add an ECA attention mechanism module (Wang et al., 2020) to the Neck structure to enhance the model’s ability to extract deep semantic feature information of detection targets and improve the accuracy of detecting incomplete targets. (3) In the Bbox-Loss module of Head, the WIoUv3 loss function (Tong et al., 2023) is used instead of the CIoU loss function (Zheng et al., 2020) to improve the convergence speed of the bounding box loss function and reduce the convergence value of the loss function. The network structure is shown in **Figure 6**, and this algorithm is named ODConv_C2f-ECA-WIoU-YOLOv8n, abbreviated as the OEW-YOLOv8n model.

#### 2.5.1 ODConv dynamic convolution module

The full name of ODConv is Omni Dimensional Dynamic Convolution, also known as Full Dimensional Dynamic Convolution. This method was first introduced in the article by Li et al. (2022). The motivation for introducing this module in this article is as follows:

In YOLOv8n, the standard Conv is adopted by the C2f module, which essentially uses the same static convolution kernel to learn from the input samples in each convolution layer. At this point, the extraction of sample feature information is insufficient. To address this, researchers have studied dynamic convolutions, such as DyConv (Chen et al., 2020) and CondConv (Yang et al., 2019). The essence of dynamic convolution is to introduce an attention mechanism that learns a linear combination of *n* convolutional kernels with attention as weights. For example, the DyConv is defined as:


(3) $$y=(\alpha_{w1}W_{1}+\ldots +\alpha_{wn}W_{n})*x$$

where $x\in {\text{R}}^{h\times w\times c_{in}}$ and $y\in {\text{R}}^{h\times w\times c_{out}}$ represent the input feature and output feature respectively. *h* and *w* represent the height and width of the channels, respectively. *c_in_* and *c_out_* represent the number of input channels and output channels, respectively. *W_i_* represents the *i*th convolutional kernel. *α_wi_* is an attention scalar that weights *W_i_*, where *i* = 1,…, *n*, and * represents the convolution operation. Research has shown that dynamic convolution can effectively improve the accuracy of lightweight convolutional neural networks while ensuring efficient inference (Yang et al., 2019; Chen et al., 2020; Li et al., 2022).

However, dynamic convolution only improves the convolution operation in terms of the number of convolutional kernels, neglecting the spatial kernel size dimension, input channel dimension, and output channel dimension. This reduces the ability of the convolution operation to extract information about sample characteristics. To address this limitation, Li et al. (2022) proposed ODConv based on multi-dimensional attention mechanisms and parallel strategies, defined as:


(4) $$\begin{array}{c} y=(\alpha_{w1}⨀\alpha_{f1}⨀\alpha_{c1}⨀\alpha_{s1}⨀W_{1}+\ldots \\ +\alpha_{wn}⨀\alpha_{fn}⨀\alpha_{cn}⨀\alpha_{sn}⨀W_{n})*x \end{array}$$

**Figure 7:**
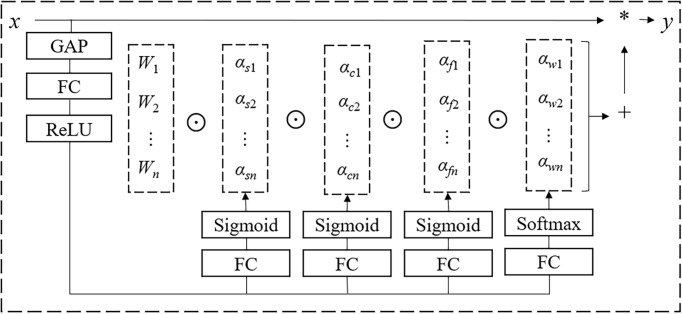
ODConv structure.

*x* represents the input features, while *y* represents the output features. *W_i_* represents the *i*th convolutional kernel. *α_wi_*, *α_fi_*, *α_ci_*, and *α_si_* are four attention scalars, respectively, representing the weights of the convolutional kernel *W_i_* (*i* = 1, …, *n*) in the dimensions of kernel quantity, output channels, input channels, and spatial kernel size. The structure is shown in **Figure 7**, where ⨀ represents the multiplication operator across different dimensions in the kernel space, and * represents the convolution operation.

In brief, in ODConv, *α_si_* assigns different attention to the filters (convolution parameters) at each spatial location (*k* × *k* in total, where *k* is the kernel size). *α_ci_* assigns different attention to each input channel (*C_in_*). *α_fi_* assigns different attention to each output channel (*C_out_*). *α_wi_* assigns different attention to each convolutional kernel. These four types of attention are complementary and are multiplied with the convolutional kernel *W_i_* in a sequential order of position, channel, filter, and kernel. This provides performance guarantees for capturing rich temporal and spatial cues. In summary, compared to mainstream dynamic convolutions like DyConv and CondConv, ODConv enhances feature learning capabilities and improves the accuracy of convolutional neural networks by obtaining complementary attention for the convolutional kernels along all dimensions of the kernel space in each convolutional layer. Additionally, ODConv outperforms other attention modules in adjusting output features or convolutional weights.

Considering that the Backbone network and Neck network of the YOLOv8n model mainly rely on the C2f module for connection, the main function of the C2f module is to input the multi-scale feature maps extracted by the Backbone network into the Neck network. To this end, the ODConv module is introduced into the C2f module that connects to the Neck network, which will fully exploit the target feature information across different dimensions. This allows richer image features to be transferred from the Backbone network to the Neck network, thereby enhancing the model’s ability to recognize small and partially occluded targets. Subsequent experiments will show that not only is the model’s performance significantly improved, but also the computational complexity is reduced during the training process.

#### 2.5.2 ECA attention mechanism

The seedling soil used for hybrid rice seedling nursing is usually a mixture of peat, vermiculite, coconut coir, clay, specialized fertilizer, and yellow soil. Owing to the different colors and shapes of the components in the seedling soil, a complex background is formed in the seed images of hybrid rice, which interferes with the identification of the number of seeds in a unit seed grid image. In addition, the unit seed grid image is generated by cutting the seed image, and some seeds are cut into incomplete seeds, which also poses some difficulties for accurate seed counting. To improve the model’s focus on seeds, this paper introduces the ECA attention mechanism (Wang et al., 2020) into the Neck network of the model (the introduced location is shown in **Figure 6**). This mechanism enables the network to prioritize the semantic information in the feature map, thereby enhancing the network’s ability to extract deep semantic feature information of detection targets. Thus, the model can better obtain the feature information of seed images while suppressing background information such as seedling soil.

**Figure 8:**
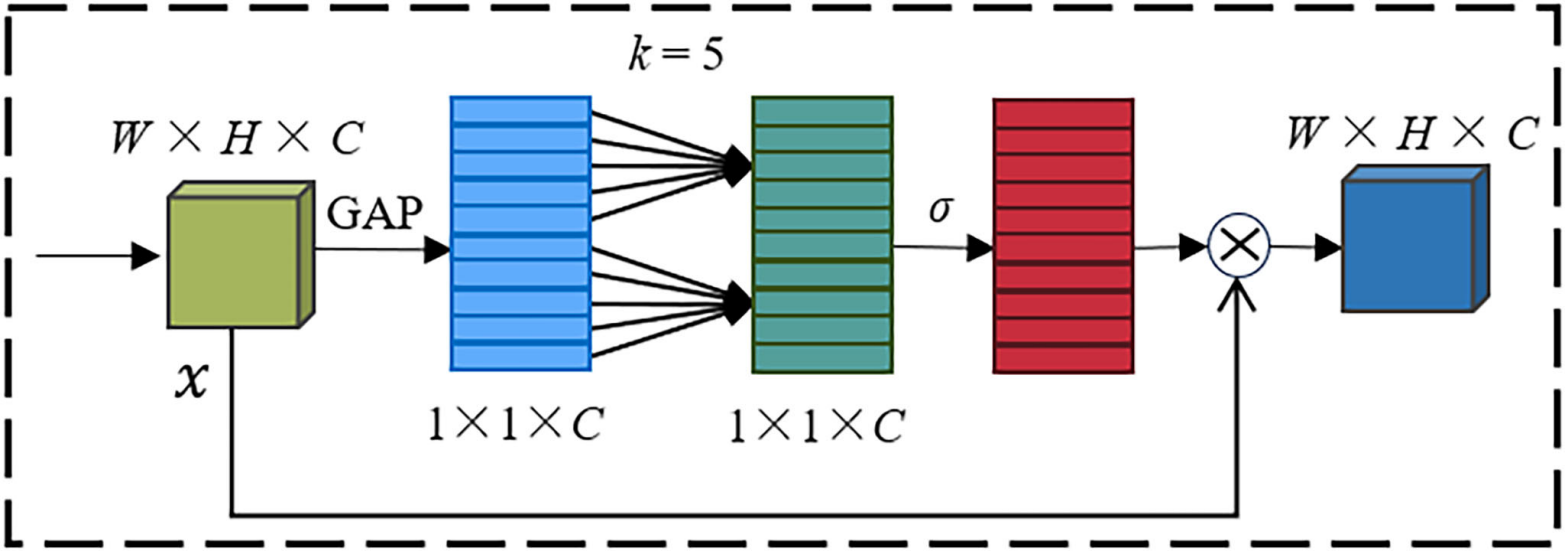
ECA module.

In neural networks, attention mechanisms are typically classified into spatial attention mechanisms and channel attention mechanisms. The channel attention mechanism assigns different weights to the channels of feature maps, allowing the model to have different degrees of attention to different channels of the feature map, thereby capturing local important feature information, suppressing interference feature information, and improving recognition accuracy. ECA is a type of channel attention mechanism that has been improved from the traditional Squeeze and Excitation Networks (SE) attention mechanism. The main improvements include (1) removing the fully connected layers in the SE attention mechanism module; (2) using a one-dimensional convolution to learn features after global average pooling (GAP); and (3) proposing a non-dimensional reduction local cross-channel interaction strategy, effectively avoiding the impact of dimensionality reduction on the learning effect of channel attention. Its module structure is shown in **Figure 8**.

In **Figure 8**, *x* represents the input features with dimensions *H*×*W* ×*C*. After global average pooling (GAP), *x* is compressed into a feature vector of size 1×1×*C*. A one-dimensional convolution operation is performed on the feature vector, where the kernel size *k* is adaptively determined. The weights of each channel are computed through a Sigmoid activation function, denoted as *σ* in **Figure 8**. Multiply the weighted feature vector with the input features *x* to get the output features $\tilde{x}$ , and in **Figure 8**, ⊗ denotes the multiplication.

#### 2.5.3 WIoU loss function

**Figure 9:**
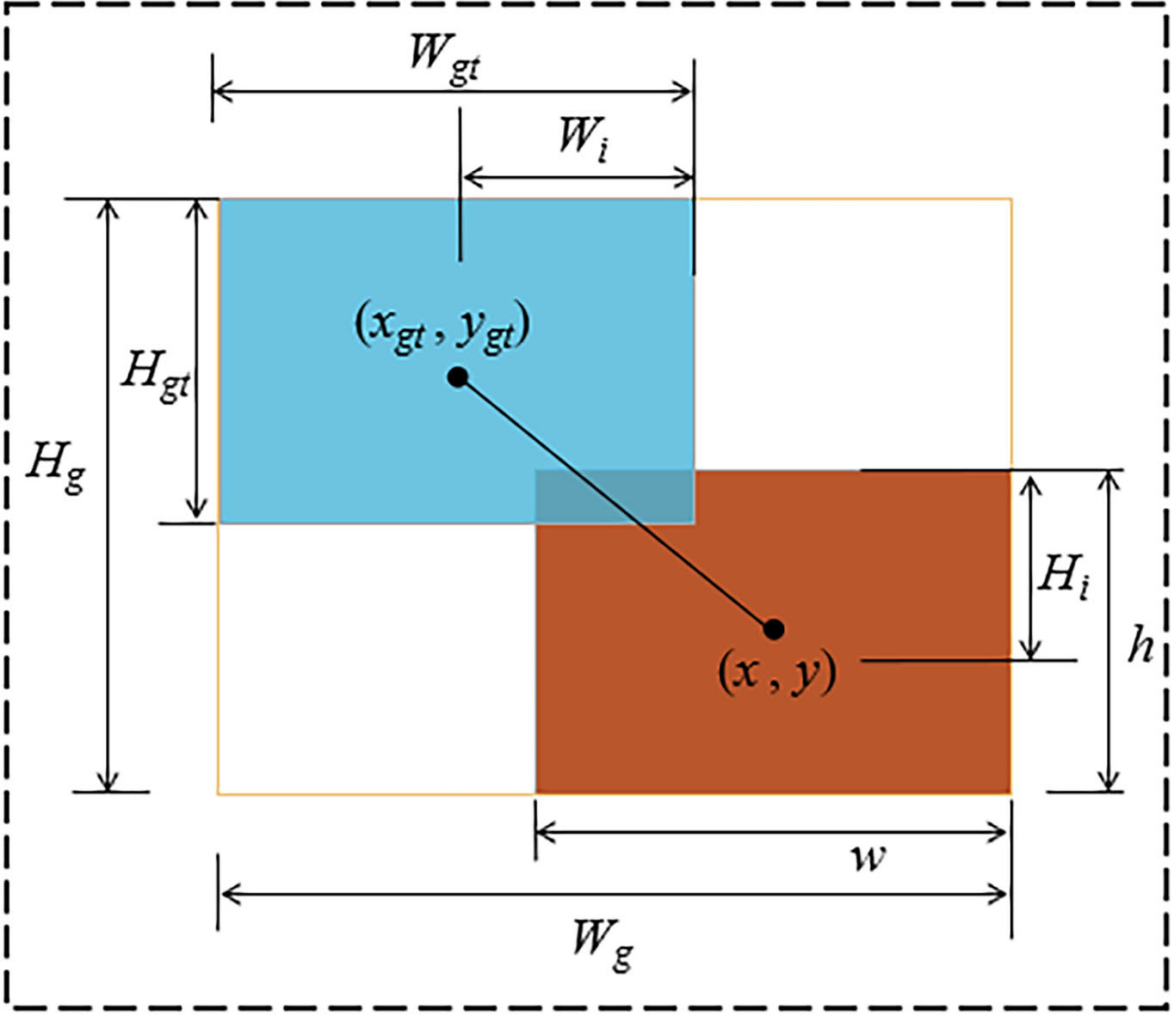
Illustration of ground truth boxes, predicted boxes, and their intersection over union.

YOLOv8n’s loss function consists of two parts: classification loss and regression loss. The classification loss is computed using BCE Loss, while the regression loss is computed using CIoU Loss. The formula for calculating CIoU Loss is as follows:


(5) $$L_{\text{CloU}}=L_{\text{IoU}}+\frac{{\left(x-x_{gt}\right)}^{2}+{\left(y-y_{gt}\right)}^{2}}{\left(W_{g}^{2}+H_{g}^{2}\right)}+\alpha v$$

(6) $$\alpha =\frac{v}{L_{\text{IoU}}+v}$$

(7) $$v=\frac{4}{\pi^{2}}{\left(\text{arctan}\frac{w}{h}-\text{arctan}\frac{w_{gt}}{h_{gt}}\right)}^{2}$$

(8) $$L_{\text{IoU}}=\frac{W_{i}H_{i}}{wh+w_{gt}h_{gt}-W_{i}H_{i}}$$

where *w*, *h*, and (*x*, *y*) represent the width, height, and center coordinates of the predicted bounding box, respectively; *w_gt_*, *h_gt_*, and (*x_gt_*, *y_gt_*) represent the width, height, and center coordinates of the ground truth bounding box, respectively; *W_g_* and *H_g_* represent the width and height of the smallest enclosing rectangle that contains both the ground truth and predicted bounding boxes; *W_i_* and *H_i_* denote the width and height of the intersection area between the ground truth and predicted bounding boxes. *α* is a weighting function used to balance the parameters, and *v* is a parameter used to measure the consistency of the length–width ratio, as shown in **Figure 9**.

Although the CIoU loss function considers overlapping area, distance, and aspect ratio, CIoU employs a monotonic focusing mechanism. This means that the bounding box regression loss uses fixed weights to balance the losses of position and scale. Because of the influence of geometric factors such as distance and aspect ratio, this can increase the penalty on low-quality samples and reduce the generalization performance of the YOLOv8n model. Specifically, when the aspect ratio of the predicted box and the ground truth box are linearly related, the penalty term of CIoU degrades to zero. In this case, whether the anchor boxes are of high quality or low quality, it can be detrimental to the regression loss (Tong et al., 2023).

Owing to the artificial cutting of some seeds in the unit seed grid image, there are some low-quality anchor boxes. Therefore, this paper chooses the dynamic non-monotonic focusing mechanism of the WIoU loss function to replace the original CIoU loss function. Essentially, the WIoU loss function adaptively adjusts the weights of regression loss based on the importance of the targets by introducing an attention mechanism. This helps to better balance the losses of position and scale, thereby balancing low-quality and high-quality samples to improve the accuracy of object detection. There are currently three versions of WIoU: WIoUv1, WIoUv2, and WIoUv3, among which WIoUv2 and WIoUv3 are enhanced versions of WIoUv1 (Tong et al., 2023). WIoUv3 is used in this paper, which is defined as follows:


(9) $$L_{\text{WIoUv}3}=rL_{\text{WIoUv}1},r=\frac{\beta }{\delta \alpha^{\beta -\delta }}$$

(10) $$\beta =\frac{{\tilde{L}}_{\text{IoU}}}{L_{\text{IoU}}}\in [0,+\infty )$$

(11) $$L_{\text{WIoUv}1}=R_{\text{WIoU}}L_{\text{IoU}}$$

(12) $$R_{WIoU}=\text{exp}(\frac{{\left(x-x_{gt}\right)}^{2}+{\left(y-y_{gt}\right)}^{2}}{{\left(W_{g}^{2}+H_{g}^{2}\right)}^{*}})$$

where *r* represents the non-monotonic focusing coefficient, *β* represents the outlier degree, and *α* and *δ* represent hyperparameters, which are set to 1.9 and 3, respectively. $\bar{L_{\text{IoU}}}$ denotes the moving average, and ${\tilde{L}}_{\text{IoU}}$ indicates an operation that separates from the computation graph, making it a constant without gradients. When $R_{\text{WIoU}}\in [1,e)$ , it increases the *L*_IoU_ of medium-quality anchor boxes, and when $L_{\text{IoU}}\in [0,1]$ , it reduces the *R*_WIoU_ of high-quality anchor boxes.

WIoUv3 uses the outlier degree *β* instead of the overlap area IoU to evaluate the quality of the anchor box. Since the moving average is dynamic, the standard for classifying anchor box quality is also dynamic. The non-monotonic focusing coefficient *r* is constructed from the outlier degree and hyperparameters. It can reduce the gradient gain of the loss function on high-quality samples and decrease the harmful gradients generated by low-quality anchor boxes, allowing WIoUv3 to focus on medium-quality anchor boxes.

### 2.6 Evaluation methods for sowing uniformity

#### 2.6.1 Determination of which seed grid the seed belongs to

After dividing the seed images into unit seed grid images, the seeds located on the boundaries of the seed grids may encounter the problem of misjudging the ownership of the seed grid.

**Figure 10:**
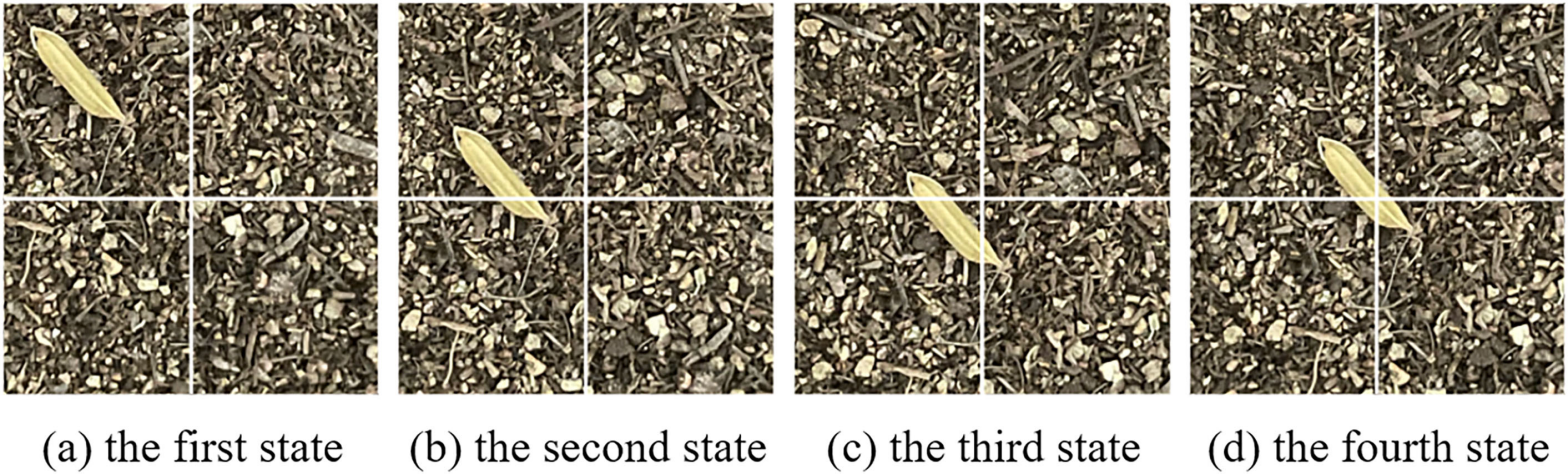
The positional relationship between seeds and seed grids. **(A)** the first state, **(B)** the second state, **(C)** the third state, and **(D)** the fourth state.

Overall, there are four situations regarding the positional relationship between seeds and seed grids (as shown in **Figure 10**): (a) Seeds do not intersect with the boundaries of seed grids, and seeds completely fall within a certain seed grid; (b) the seed intersects with one seed grid boundary and falls into two adjacent seed grids; (c) the seeds intersect with the boundaries of two seed grids, and the seeds fall into three adjacent seed grids; (d) the seeds intersect with the boundaries of the four seed grids, and the seeds fall within the adjacent four seed grids.

At present, there is no good way to determine which seed grid a seed belongs to when there is a cross between the seed and the seed grid. In this paper, an area-based approach is employed to determine the ownership of seeds within seed grids. Specifically, the seed grid with the largest proportion of the area of the seed in the adjacent four seed grids is considered as the seed’s belonging grid. The specific method is as follows: According to the OEW-YOLOv8n model provided in the following text, the seed recognition box covers the area size within four adjacent seed grids for discrimination, and the seed grid with the largest coverage area is the seed grid where the seed belongs.

#### 2.6.2 Evaluation methods for uniformity

The key to evaluating the uniformity of seed images using uniform qualification rate is to calculate the number of qualified seed grid images for each seed image. According to the national standard of the People’s Republic of China “Rice Transplanter-Test Method” (GB/T 6243-2017), the uniformity qualification rate is calculated as follows: the uniformity qualification rate = (number of qualified small seedling blocks/total number of seedling blocks tested) × 100%. Because of the agronomic requirement of planting one to three seedlings per hill in hybrid rice cultivation, this article uses the qualification rate of one to three seeds per grid as the evaluation indicator of sowing uniformity. The qualification rate of one to three seeds per grid is calculated as: The qualification rate of one to three seeds per grid = (number of grids with one to three seeds per grid ÷ total number of seed grids per tray) × 100%.

## 3 Results

### 3.1 Training environment and methods

Using the above method, we trained unit seed grid images. The dataset comprises a total of 23,814 images. Models such as OEW-YOLOv8n were used, with the input image resolution adjusted to 640 × 640 pixels. In order to adapt to the convergence effect of the model and the complexity of the dataset, The optimizer employed for training was Stochastic Gradient Descent (SGD). The number of workers was set to 2, and the batch size was set to 32. The initial learning rate was 0.01, and the training lasted for 200 epochs. The model training and testing environment included a 64-bit Windows 10 Professional operating system, a 13^th^-generation Intel(R) Core (TM) i5-13600KF CPU running at 3.5 GHz, 32 GB of RAM, an NVIDIA GeForce RTX 2070 SUPER GPU with 8 GB of VRAM, CUDA version 11.7, the deep learning framework PyTorch1.12, and Python version 3.8.

### 3.2 Evaluating indicator

In this research, Params, floating point operations (FLOPs), and model size are used to reflect the complexity of a model, where FLOPs are used to measure computational amount. Precision (*P*), recall (*R*), and mAP [as described in Equations 13–16] are used to evaluate the detection performance of a model. Root mean square error (RMSE) is used to quantify the deviation between the model’s estimated value and true value, with a lower score indicating less error and better model performance.


(13) $$P=\frac{T_{P}}{T_{P}+F_{p}}\times 100\%$$

(14) $$R=\frac{T_{P}}{T_{P}+F_{N}}\times 100\%$$

(15) $$AP=\int_{0}^{1}P\text{d}R\times 100\%$$

(16) $$mAP=\frac{1}{N}\sum_{i=1}^{N}AP(i)$$

In Equations 13–16, *T_p_* and *F_p_* represent true-positive and false-positive instances, respectively. *F_n_* represents false-negative instances. *AP*(*i*) denotes the average precision for the *i*th class, and *N* represents the total number of classes. In this paper, *N* equals 1, as there is only one class, which is the seed class.


(17) $$RMSE=\sqrt{\frac{1}{M}\sum_{i=1}^{M}{\left(y_{i}^{pred}-y_{i}^{gt}\right)}^{2}}$$

where $y_{i}^{pred}$ represents the estimated value, $y_{i}^{\text{g}t}$ represents the true value, and *M* represents the number of samples.

### 3.3 The detection results of seed grid and seed count based on the OEW-YOLOv8n model

#### 3.3.1 Ablation experiments

The results of the ablation experiment are shown in **Table 3**.

**Table 3:** Ablation experiment results.

Number	ODConv	ECA	WIoU	Params (M)	FLOPs (G)	Model size (MB)	P (%)	R (%)	mAP@0.5 (%)	RMSE
0	×	×	×	3.01	8.2	6.13	92.3	90.5	96.1	0.445
1	√	×	×	3.35	8.0	6.83	95.1	93.6	97.4	0.392
2	×	√	×	3.01	8.2	6.14	94.2	93.1	96.7	0.426
3	×	×	√	3.01	8.2	6.13	93.8	92.7	96.5	0.435
4	√	√	×	3.35	8.0	6.84	96.2	94.9	98.1	0.299
5	√	×	√	3.35	8.0	6.83	95.2	94.5	97.7	0.365
6	×	√	√	3.01	8.2	6.14	94.3	93.2	97.2	0.417
7	√	√	√	3.35	8.0	6.84	96.8	95.3	98.6	0.269

“√” indicates that the corresponding policy is used, and “×” indicates that the corresponding policy is not used.

Based on **Table 3**, it is evident that after replacing the Conv module in the YOLOv8n network with the ODConv module in Experiment 1, the Params and model size slightly increased, but the FLOPs decreased. The precision (*P*), recall (*R*), and mAP of the model increased by 2.8%, 3.1%, and 1.3%, respectively. The RMSE decreased by 12%. Experiments 2 and 3 showed that adding ECA attention mechanism or replacing CIoU loss function with the WIoUv3 loss function did not significantly change the Parms, FLOPs, and model size. The precision of the model increased by 1.9% and 1.5%, the recall increased by 2% and 2.2%, and the mAP increased by 0.6% and 0.4%, respectively. The RMSE decreased by 4% and 2%. These experiments demonstrate that both the ECA attention mechanism and the WIoUv3 loss function can enhance the model’s detection accuracy. Experiment 4 is an experiment adding the ECA attention mechanism on Experiment 1. The Params and FLOPs remained nearly unchanged, while the model size slightly increased. The precision, recall, and mAP further improved, with the mAP increasing from 97.4% to 98.1%, an increment of 0.7%. The RMSE decreased by 23.7%. Experiment 5: Building on Experiment 1 by replacing the CIoU loss function with the WIoUv3 loss function, there was no significant change in the Params, FLOPs, and model size. However, precision, recall, and mAP improved by 0.1%, 0.9%, and 0.3%, respectively. The RMSE decreased by 7%. Experiment 6: Building on Experiment 2 by replacing the CIoU loss function with the WIoUv3 loss function, the Params, FLOPs, and model size remained largely unchanged, with improvements observed in precision, recall, and mAP. The RMSE decreased by 2%. Experiment 7: Building on Experiment 4 by replacing the CIoU loss function with the WIoUv3 loss function, the Params, FLOPs, and model size remained largely unchanged. Precision, recall, and mAP improved by 0.6%, 0.4%, and 0.5%, respectively. The RMSE decreased by 10%.

In summary, using the ODConv module to replace the original Conv module slightly increases the Params and model size, but reduces the FLOPs by 0.2 G and improves the mAP by 1.3%. Adding the ECA attention mechanism does not significantly change the model’s complexity, yet it raises the model’s mAP by approximately 0.6%. Replacing the CIoU loss function with the WIoUv3 loss function also does not alter the model’s complexity, but the precision improves by 0.4%. Implementing all three strategies simultaneously results in an increase of 0.34 M in Params, 0.71 MB in model size, a reduction of 0.2 G in FLOPs, and improvements in precision, recall, and mAP by 4.5%, 4.8%, and 2.5%, respectively. This indicates that all three improvement strategies enhance the model’s detection accuracy and have a cumulative effect, with the ODConv having the most significant impact. The slight increase in Params and model size is primarily due to the introduction of the ODConv module.

**Figure 11:**
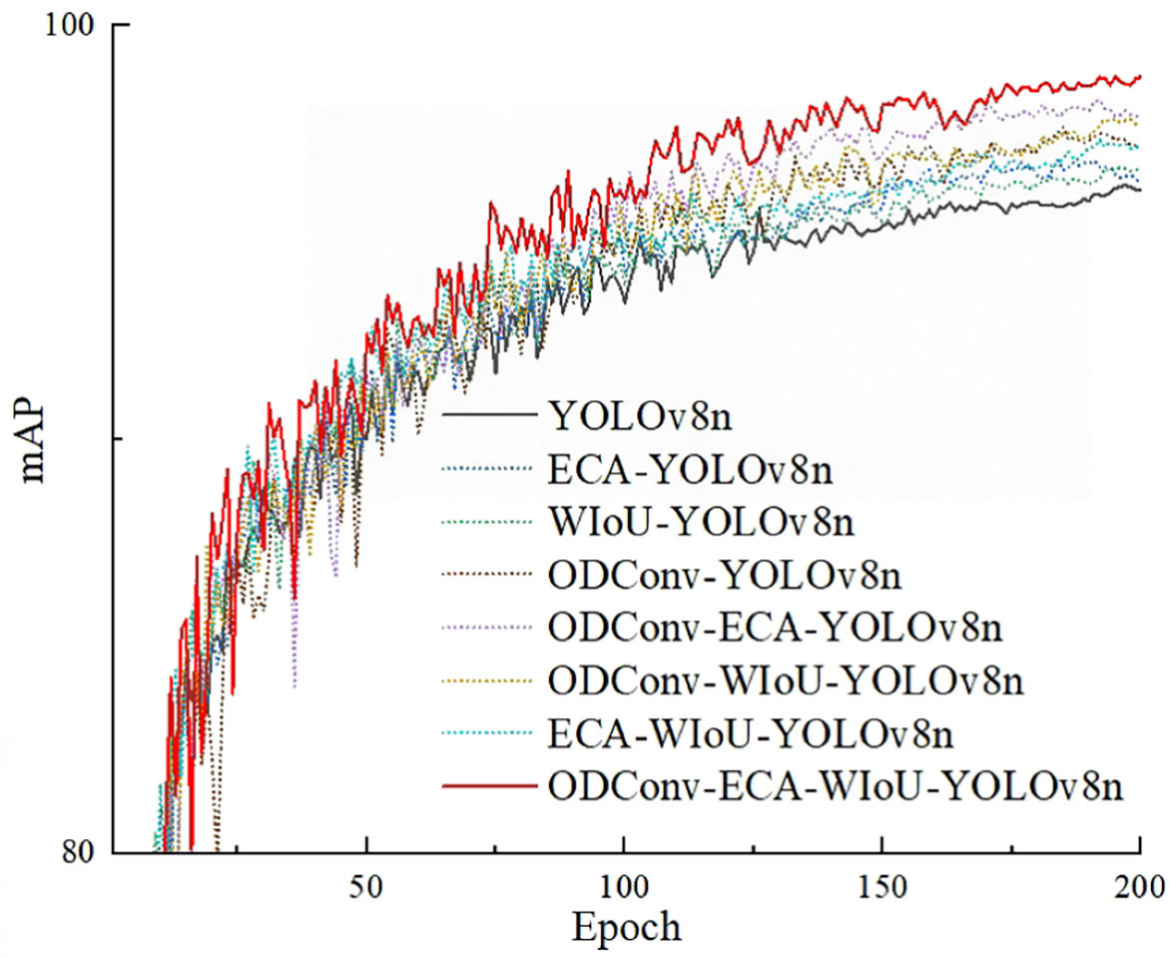
The curve of mAP.

To examine the impact of different strategies on the convergence speed of the model, **Figure 11** shows the curve of mAP of different models in the ablation experiment as the number of model iterations changes.

From **Figure 11**, it can be found that after approximately 25 iterations, the fluctuations in the mAP curve begin to diminish, and after approximately 150 iterations, the mAP curve tends to stabilize. This indicates that all models exhibit good convergence speed. Among them, Experiment 7 performs the best, while Experiment 0 performs the worst. This demonstrates that all three improvement strategies enhance convergence speed, and employing all three strategies simultaneously can achieve relatively faster convergence speeds and higher convergence values.

#### 3.3.2 Comparisons of OEW-YOLOv8n performance under different loss functions

**Figure 12:**
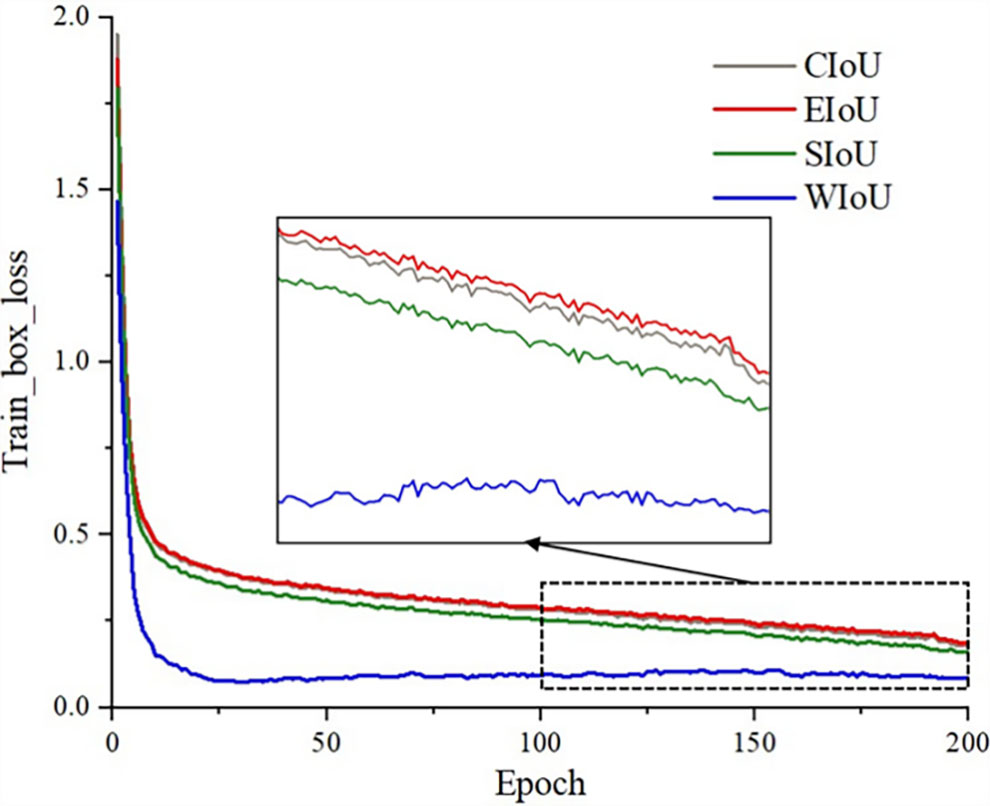
Bounding box loss.

In this study, the bounding box regression loss function has a significant impact on the accurate identification of the number of seeds in a unit seed grid. In existing deep learning models, common regression loss functions include CIoU, EIoU, SIoU, and WIoU. The loss function of the YOLOv8n model is CIoU. In order to investigate the influence of different regression loss functions on the performance of the OEW-YOLOv8n model, the convergence of OEW-YOLOv8n under different loss functions was compared and analyzed. The experimental results are illustrated in **Figure 12**.

As shown in **Figure 12**, when the bounding box regression loss function adopts EIoU, the convergence speed of the model is the slowest, and the final loss value after convergence is the highest. When using the CIoU loss function, its convergence speed and final loss value are almost equivalent to EIoU (shown in the graph as the red and gray lines overlapping). The convergence speed of using SIoU is slightly higher than that of EIoU and CIoU, and the final loss value after convergence is slightly lower than EIoU and CIoU. When WIoU is adopted, the convergence speed of the model is the fastest, converging approximately after 25 iterations. Although there are some fluctuations in the final loss value after convergence, which may be related to the use of non-monotonic focusing mechanism, the final loss value after convergence is significantly lower than those of the other three loss functions. The ablation experiment in Section 3.3.1 also showed that the use of WIoU resulted in some improvement in mAP. Therefore, this paper adopts WIoU to replace the CIoU loss function.

#### 3.3.3 Model comparison

To evaluate the performance of the OEW-YOLOv8n model, comparative experiments were conducted with advanced object detection networks such as Faster-RCNN, SSD, YOLOv4, YOLOv5s, YOLOv7-tiny, and YOLOv10s. The dataset used in these experiments is the seed grid images used in this study. The experimental results are shown in **Table 4**.

**Table 4:** Comparison of seed number detection results of different models.

Models	mAP@0.5 (%)	Params (M)	FLOPs (G)	Model size (MB)	RMSE
Faster-RCNN	93.4	136.68	199.3	110.76	0.600
SSD	90.8	23.61	273.2	92.78	0.733
YOLOv4	93.7	63.94	59.9	250.25	0.590
YOLOv5s	95.8	6.83	15.9	14.73	0.514
YOLOv7-tiny	95.7	6.02	13.2	12.32	0.544
YOLOv10s	95.3	7.19	21.6	14.69	0.567
OEW-YOLOv8n	98.6	3.35	8.0	6.84	0.269

According to **Table 4**, the Params, FLOPs, model size, and RMSE of the OEW-YOLOv8n model are all smaller than those of the other five models. Compared to Faster-RCNN, SSD, YOLOv4, YOLOv5s, YOLOv7-tiny, and YOLOv10s, the Params of the OEW-YOLOv8n model is reduced by 133.33 M, 20.26 M, 60.59 M, 3.48 M, 2.67 M, and 3.84 M, respectively. The FLOPs are reduced by 191.3 G, 265.2 G, 51.9 G, 7.9 G, 5.2 G, and 13.6 G, respectively, and the model size is reduced by 103.92 MB, 85.94 MB, 243.41 MB, 6.89 MB, 5.19 MB, and 7.85 MB, respectively. These indicate that the complexity of the model has significantly decreased. Conversely, the mAP of the OEW-YOLOv8n model is higher than that of the other five models, with improvements of 5.2%, 7.8%, 4.9%, 2.8%, 2.9%, and 3.3% over Faster-RCNN, SSD, YOLOv4, YOLOv5s, YOLOv7-tiny, and YOLOv10s, respectively. The RMSE is reduced by 0.331, 0.464, 0.321, 0.245, 0.275, and 0.298, respectively. Overall, the two-stage network model Faster-RCNN has high model complexity and low recognition accuracy. The one-stage network models such as SSD, YOLOv4, YOLOv5s, YOLOv7-tiny, and YOLOv10s have a relatively lower model complexity and a higher recognition accuracy.

**Figure 13:**
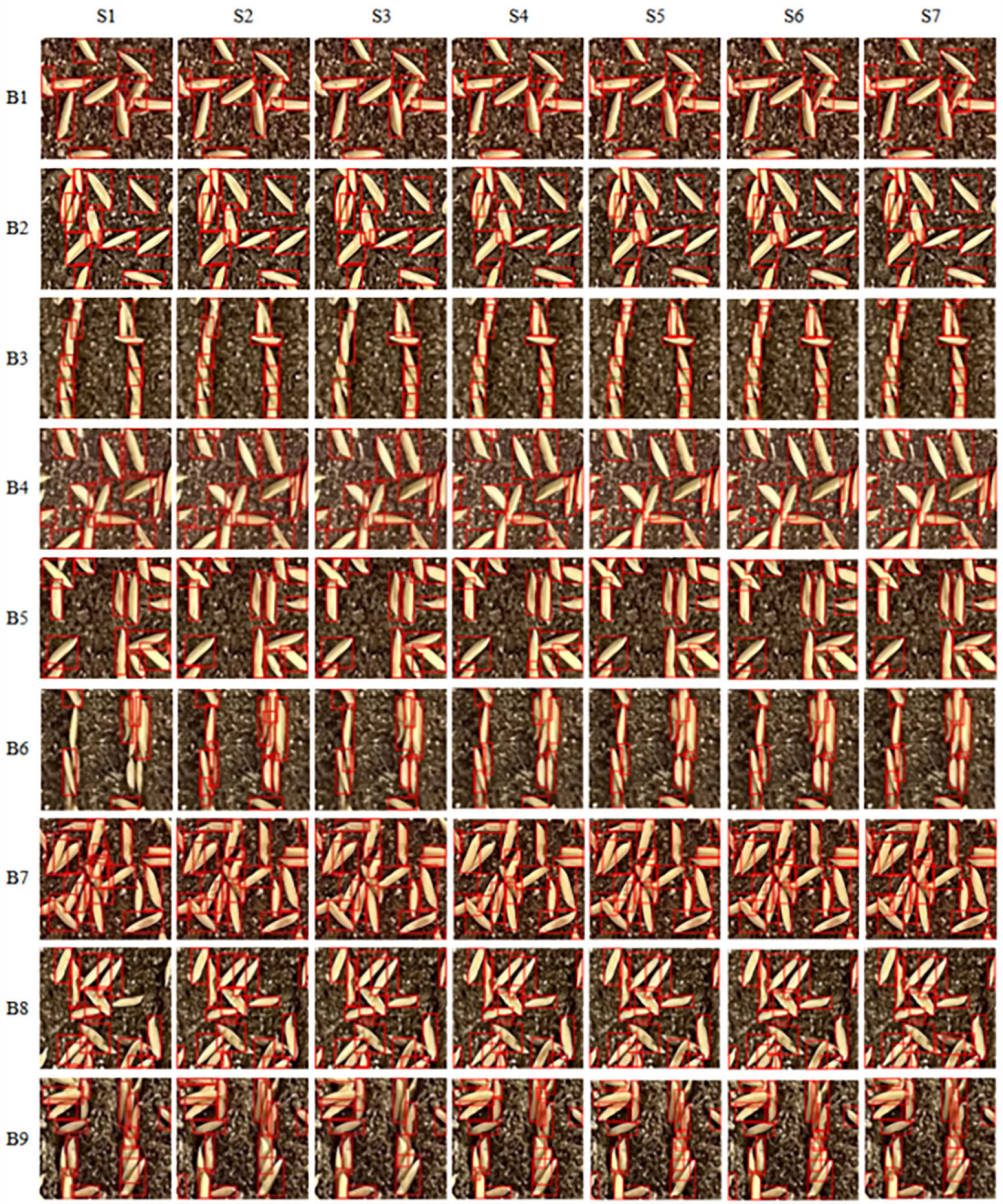
Comparison of detection performance of different object detection models. S1–S7 represent the SSD, Faster-RCNN, YOLOv4, YOLOv5s, YOLOv7-tiny, YOLOv10s, and OEW-YOLOv8 algorithms, respectively, while B1–B9 represent broadcast sowing with 50 g/tray, no-ditching drill sowing with 50 g/tray, ditching drill sowing with 50 g/tray, broadcast sowing with 60 g/tray, no-ditching drill sowing with 60 g/tray, ditching drill sowing with 60 g/tray, broadcast sowing with 70 g/tray, no-ditching drill sowing with 70 g/tray, and ditching drill sowing with 70 g/tray, respectively.

To analyze the reasons for the differences in detection performance among different object detection models, **Figure 13** shows the detection results of a representative seed grid under different sowing densities and sowing methods. In the experiment, to increase the number of seeds and reduce the segmentation of seeds during seed grid division and test the generalization ability of the model, a seed image was divided into 144 seed grids according to a specification of 9 times by 16 times in the experiment.

From **Figure 13**, it can be seen that the SSD model performs the worst, with a large number of missed detections. For example, in cell B3, there are 13 seeds in the image (with 11 actual effective seeds), but the SSD model only identifies 8 seeds, missing 5 seeds, resulting in a 38.46% miss rate. In the SSD model, the missed seeds are mainly defective seeds at the edges of the image or seeds that are obscured. While Faster-RCNN, YOLOv4, YOLOv5s, YOLOv7-tiny, and YOLOv10s can identify defective seeds at the image edges, they also exhibit missed detections and have poor recognition accuracy for seeds obscured by seedling soil. The OEW-YOLOv8n model performs very well overall, capable of identifying both defective seeds at the image edges and obscured seeds, with almost no missed detections.

In summary, compared to the existing advanced networks, the improved OEW-YOLOv8n model performs better in detecting the number of seeds per unit seed grid.

### 3.4 Evaluation experiment of sowing uniformity in hybrid rice blanket-seedling nursing

To investigate the practical application effect of the algorithm on the evaluation of sowing uniformity, a comparative experiment was conducted between the evaluation of sowing uniformity algorithm and manual evaluation, with uniform qualification rate and empty grid rate as evaluation indicators. The evaluation algorithm adopts the method described in Section 2 of this article. The manual evaluation adopts the method of manually counting the number of seeds per unit grid, as follows:

According to the method described in **Table 1**, seedling trays with 50 g/tray, 60 g/tray, and 70 g/tray were divided into 738 grids/tray, 864 grids/tray, and 1,044 grids/tray, respectively. In the experiment, the grid frame was made of transparent thin wires, and the grid frame specifications for 50 g/tray, 60 g/tray, and 70 g/tray were 15.56 mm × 14.15 mm, 15.56 mm × 12.08 mm, and 15.56 mm × 10.00 mm, respectively. These grid frames were used to divide the seed trays into seed grids. Then, the number of seeds in each seed grid was counted. The uniformity qualification rate was calculated according to the method in Section 2.6.2, and the empty grid rate was calculated as follows: empty grid rate = (number of empty grid/total number of grids) × 100%. For each type of seedling trays, three trays were tested, which is equivalent to three repetitions. The average of the results of the three tests was taken as the corresponding test result. The results are shown in **Table 5**.

**Table 5:** Sowing uniformity evaluation test results.

Seedling density/(g·tray−1)	Seedling type			Testing empty grid rate (%)	Actual empty grid rate (%)	Testing uniformity qualification rate (%)	Actual uniformity qualification rate (%)	Test error (%)
	Broadcast sowing	No-ditching strip sowing	Ditching strip sowing					
50	√	×	×	4.67	4.82	68.75	70.88	2.13
	×	√	×	4.30	4.26	72.63	75.47	2.84
	×	×	√	3.75	3.54	82.77	80.82	−1.95
60	√	×	×	3.82	3.79	70.83	69.27	−1.56
	×	√	×	3.19	3.05	73.05	75.10	2.05
	×	×	√	2.77	2.72	77.91	80.83	2.92
70	√	×	×	2.75	2.62	65.97	67.22	1.25
	×	√	×	1.94	2.04	69.86	72.43	2.57
	×	×	√	1.11	1.18	79.72	77.29	−2.43

In **Table 5**, the empty grid rate and uniformity qualification rate resulting from algorithm evaluation are respectively named as testing empty grid rate and testing uniformity qualification rate, and the empty grid rate and uniformity qualification rate resulting from manual evaluation are named actual empty grid rate and actual uniformity qualification rate, respectively. Test error = actual uniformity qualification rate − testing uniformity qualification rate.

According to **Table 5**, the experiment indicates errors of −0.15% to 0.21% for empty grid rate and −2.43% to 2.92% detection, indicating that the algorithm has good accuracy. The actual uniformity qualification rates of ditching strip sowing with 50 g/tray, 60 g/tray, and 70 g/tray are 80.82%, 80.83%, and 77.29%, respectively, with an average of 79.65%. The actual uniformity qualification rates of no-ditching drill sowing with 50 g/tray, 60 g/tray, and 70 g/tray are 75.47%, 75.10%, and 72.43%, respectively, with an average of 74.33%. The actual uniformity qualification rates of broadcast sowing with 50 g/tray, 60 g/tray, and 70 g/tray are 70.88%, 69.27%, and 67.22%, respectively, with an average of 69.12%. From this, it can be seen that the effect of sowing density on sowing uniformity is relatively small when the sowing density is not too high. Among different sowing methods, the order of uniformity qualification rates from high to low is as follows: ditching strip sowing > no-ditching strip drill sowing > broadcast sowing, which is consistent with the actual situation.

## 4 Discussion

(1) The evaluation method proposed in this paper is mainly suitable for evaluating the sowing uniformity of rice under low-density conditions (usually referring to sowing density less than 70 g/tray). On one hand, according to the agronomic requirements of rice production (five to seven seedlings per hill for conventional rice and one to three seedlings per hill for hybrid rice), high-density sowing is usually used for conventional rice seedling nursing, while low-density sowing is used for hybrid rice seedling nursing. When high-density sowing is used for seedling nursing, the impact of sowing uniformity on the quality of rice transplanting is relatively small; however, when using low-density sowing for seedling nursery, the sowing uniformity has a significant impact on the quality of rice transplanting. Therefore, the evaluation of rice sowing uniformity is mainly applied in low-density sowing for hybrid rice seedling nursing. On the other hand, when the sowing density is very high, such as more than 120 g/tray, there will be a lot of overlapping seeds in the seedling tray. It is very difficult to evaluate sowing uniformity whether manually or through machine vision, and its evaluation significance is also very limited. As a result, this paper does not involve the evaluation in high-density sowing condition, and the impact of seed overlapping on the algorithm has not been considered temporarily.

(2) Assuming that one grid in a blanket tray is equivalent to one pot in a pot tray, we can find that the detection method adopted by Tan et al. (2014) achieved a mAP 94.4%, while our research method achieved 98.6%, an improvement of 4.2 percentage points. Dong et al. (2020) proposed a detection method with an mAP of 90.98%, whereas our research method achieved 98.6%, an improvement of 7.62 percentage points.

(3) In the detection area of this article, a seedling tray includes 738–1,044 grids. In terms of detection time, the average detection time is 7 ms per unit seed grid image. Compared to the detection method proposed by Tan et al. (2014), the detection time per grid image is reduced from 10.28 to 7 ms, a decrease of 3.28 ms.

(4) In practical applications, the detection of sowing uniformity is mainly applied in two places: first, evaluating the performance testing of seeders carried out by institutions; the second is to adjust the performance of the seeder during production to ensure the stability of sowing quality. Objectively speaking, the method proposed in this article can adapt well to the first scenario, but it still cannot adapt well to the second scenario. There are still some difficulties in deploying this method to achieve real-time detection on edge devices such as smartphones. This is a limitation of the evaluation method proposed in this paper. In the future, we will research how to achieve real-time online detection without reducing detection accuracy. In addition, to meet the needs of large-scale farm production, we will conduct systematic research on the impact of factors such as lighting environment, variety, and image acquisition equipment on the detection effect in our subsequent work.

## 5 Conclusions

In response to the issue of evaluation of sowing uniformity in hybrid rice blanket-seedling nursing, this paper proposes an evaluation method that integrates image processing with the OEW-YOLOv8n model, and an evaluation experiment for the sowing uniformity in hybrid rice blanket-seedling nursing was conducted. The following conclusions were mainly obtained:

(1) On the test set, the model achieves a mAP of 98.6%. The model size is 6.84 MB, the FLOPs are 8.0 G, and the number of parameters is 3.35 M. Compared to the original model, with a slight increase in model size and parameters, the FLOPs, precision, recall, and mAP increased by 0.2 G, 4.5%, 4.8%, and 2.5%, respectively.
(2) Among the three improvement strategies suggested, the strategy of using ODConv module has the greatest impact on model improvement. This strategy can respectively increase the precision, recall, and mAP by 2.8%, 3.1%, and 1.3%. Similarly, adding the ECA attention mechanism or replacing the CIoU loss function with the WIoUv3 loss function can increase the precision, recall, and mAP by 1.9%, 2.6%, and 0.6%, or 1.5%, 2.2%, and 0.4%, respectively. In addition, replacing the CIoU loss function with the WIoUv3 loss function has little effect on the complexity of the model, and these three improvement strategies have a cumulative effect on improving the detection performance of the model.
(3) The analysis of application sample experiments shows that the algorithm proposed in this paper has satisfactory accuracy. The experiment indicates errors of −0.15% to 0.21% for empty grid rate and −2.43% to 2.92% detection. Furthermore, the study also found that the sowing density has little impact on sowing uniformity in low-density sowing. In terms of different sowing methods, the order of sowing uniformity qualification rates from high to low is ditching strip sowing > no-ditching strip sowing > broadcast sowing, which is consistent with the actual situations.

## Data Availability

The raw data supporting the conclusions of this article will be made available by the authors, without undue reservation.
